# Sulfonation of IAA in *Urtica* eliminates its DR5 auxin activity

**DOI:** 10.1007/s00299-024-03399-1

**Published:** 2024-12-20

**Authors:** Klara Supikova, Asta Žukauskaitė, Andrea Kosinova, Aleš Pěnčík, Nuria De Diego, Lukáš Spíchal, Martin Fellner, Katerina Skorepova, Jiri Gruz

**Affiliations:** 1 Department of Experimental Biology, Palacký University Olomouc, Šlechtitelů 27, CZ-77900 Olomouc, Czech Republic; 2 Department of Chemical Biology, Palacký University Olomouc, Šlechtitelů 27, CZ-77900 Olomouc, Czech Republic; 3 Laboratory of Growth Regulators, Institute of Experimental Botany, The Czech Academy of Sciences & Faculty of Science, Palacký University, Olomouc, Czech Republic; 4 Czech Advanced Technology and Research Institute (CATRIN), Palacký University Olomouc, Šlechtitelů 27, CZ-77900 Olomouc, Czech Republic

**Keywords:** Indole-3-acetic acid, Sulfonated, *N*-Sulfoindole-3-acetic acid, Phytohormone, Mass spectrometry, Metabolomics

## Abstract

**Key message:**

*N*-Sulfonated IAA was discovered as a novel auxin metabolite in *Urtica *where it is biosynthesized *de novo *utilizing inorganic sulfate. It showed no auxin activity in DR5::GUS assay, implying possible inactivation/storage mechanism.

**Abstract:**

A novel auxin derivative, *N*-sulfoindole-3-acetic acid (IAA-*N*-SO_3_H, SIAA), was discovered in stinging nettle (*Urtica dioica*) among 116 sulfonated metabolites putatively identified by a semi-targeted UHPLC–QqTOF-MS analysis of 23 plant/algae/fungi species. These sulfometabolites were detected based on the presence of a neutral loss of sulfur trioxide, as indicated by the *m/z* difference of 79.9568 Da in the MS^2^ spectra. The structure of newly discovered SIAA was confirmed by synthesizing its standard and comparing retention time, *m/z* and MS^2^ spectrum with those of SIAA found in *Urtica*. To study its natural occurrence, 73 species in total were further analyzed by UHPLC–QqTOF-MS or targeted UHPLC–MS/MS method with a limit of detection of 244 fmol/g dry weight. However, SIAA was only detected in *Urtica* at a concentration of 13.906 ± 9.603 nmol/g dry weight. Its concentration was > 30 times higher than that of indole-3-acetic acid (IAA), and the SIAA/IAA ratio was further increased under different light conditions, especially in continuous blue light. In addition to SIAA, structurally similar metabolites, *N*-sulfoindole-3-lactic acid, 4-(sulfooxy)phenyllactic acid and 4-(sulfooxy)phenylacetic acid, were detected in *Urtica* for the first time. SIAA was biosynthesized from inorganic sulfate in seedlings, as confirmed by the incorporation of exogenous ^34^S-ammonium sulfate (1 mM and 10 mM). SIAA exhibited no auxin activity, as demonstrated by both the *Arabidopsis* DR5::GUS assay and the *Arabidopsis* phenotype analysis. Sulfonation of IAA may therefore be a mechanism for IAA deactivation and/or storage in *Urtica*, similar to sulfonation of the jasmonates in *Arabidopsis*.

**Supplementary Information:**

The online version contains supplementary material available at 10.1007/s00299-024-03399-1.

## Introduction

Indole-3-acetic acid (IAA), the main auxin, plays a pivotal role in plant physiology, participating in most developmental and physiological processes regulated by phytohormones (Zhao 2010). Its main functions are regulation of cell division, cell differentiation, root and shoot elongation, vascular development, apical dominance, tropisms, senescence and flowering (Davies 2004). Besides higher plants, IAA was also identified in various algae such as *Macrocystis*, *Botryocladia*, and *Chlorella,* where its functions are similar to those in higher plants (Tarakhovskaya, et al. 2007; Zhang and van Duijn 2014). Other known auxins include structurally quite diverse indole-3-butyric acid, phenylacetic acid (PAA) and 4-chloroindole-3-acetic acid; thus, auxin characteristic is, rather than a typical structural pattern, the ability to induce the same physiological responses as IAA (Davies 2004; Korasick, et al. 2013; Zhao 2010). Furthermore, synthetic auxins, such as 2,4-dichlorophenoxyacetic acid and 1-naphthaleneacetic acid, trigger even stronger auxin response than IAA (Korasick, et al. 2013).

Efficient auxin signaling depends on precise IAA concentrations and spatial gradients in plant tissues, which are maintained by (i) biosynthesis, (ii) transport, and (iii) metabolism (Ludwig-Muller 2011). (i) Biosynthesis of IAA is ensured by several Trp-independent and Trp-dependent pathways originating from indole-3-glycerol phosphate and Trp, respectively (Korasick, et al. 2013; Woodward and Bartel 2005). In addition, β-oxidation of indole-3-butyric acid and enzymatic cleavage of auxin conjugates are also involved in the production of IAA (Korasick, et al. 2013; Woodward and Bartel 2005). (ii) The IAA transport over long distances (e.g. between organs) is carried out predominantly by the phloem, whereas IAA transport over shorter distances (e.g. cell-to-cell and between cell compartments) is mediated by active transport through specific transporters (Zazímalová, et al. 2010). IAA transport is strongly influenced by pH, i.e. at a slightly acidic pH of 5.5, typical for the apoplast and vacuoles, IAA can freely diffuse across membranes, mainly from the extracellular space into cells (Ljung 2013; Zazímalová, et al. 2010). In contrast, at a neutral pH, typical for the cytosol, IAA dissociates into IAA^−^ ion, which can only be transported out of the cells by active transport (Ljung 2013; Zazímalová, et al. 2010). (iii) Metabolism is another mechanism of IAA homeostasis and involves modification/conjugation reactions and degradation pathways, e.g. glycosylation, oxidation, methylation, and conjugation with amino acids, peptides and proteins (Korasick, et al. 2013).

The metabolism of IAA is further distinguished based on reversibility. However, both reversible and irreversible modifications of IAA typically lead to the loss of auxin activity. Reversibly modified IAA includes modifications by glycosylation, methylation, and conjugation with amino acids, peptides and proteins, mostly serving as a storage form of IAA that can be released in its free form (Korasick, et al. 2013). Glycosylation of IAA results in the formation of its glycosylesters bound via the carboxyl group of the acetic acid side chain, e.g. IAA-glucose (IAA-Glc) and IAA-*myo*-inositol (Korasick, et al. 2013). Conjugation of IAA with amino acids results in amide-linked IAA, such as IAA-Ala, IAA-Leu, IAA-Asp and IAA-Glu (Korasick, et al. 2013). Although some of these conjugates were previously reported to be irreversible, namely IAA-Glu and IAA-Asp, this assumption has recently been refuted, showing that IAA-Glu and IAA-Asp likely serve for IAA storage (Brunoni, et al. 2023; Hayashi, et al. 2021). In addition, IAA can be conjugated with proteins and polypeptides through an amide bond, typically for storage and inactivation of IAA (Bialek and Cohen 1986; Walz, et al. 2001). Reversible derivatization of IAA also involves methylation, which produces IAA methyl ester, the storage form of IAA (Qin, et al. 2005; Zubieta, et al. 2003). The only irreversible modification of IAA is oxidation, which leads to the formation of a degradation product of IAA, oxindole-3-acetic acid (oxIAA), being mediated by dioxygenase for auxin oxidation 1 (DAO1) in *Arabidopsis* (Porco, et al. 2016). IAA conjugates such as IAA-Asp and IAA-Glu can also be oxidized by this enzyme and potentially can further dissociate to free oxIAA (Brunoni, et al. 2023; Hayashi, et al. 2021).

Based on the results of this study, sulfonation is proposed as another modification reaction in IAA metabolism. In general, sulfonation increases the polarity and water solubility of molecules which can dissociate to the anionic form at neutral and slightly acidic pH (Yi, et al. 2006). Various primary and secondary metabolites such as polysaccharides, peptides, proteins, steroids, flavonoids, phenylpropanoids and phytohormones have been reported as possible substrates for sulfonation^1^ (Koprivova and Kopriva 2016). The best-known sulfometabolites from plants are glucosinolates, which serve as defense molecules against herbivores and pathogens (Koprivova and Kopriva 2016). Physiological activities have also been discovered for sulfonated flavonoids, which can positively influence plant auxin efflux (Faulkner and Rubery 1992). Non-sulfonated flavonoids (e.g. quercetin) can inhibit auxin efflux, resulting in the accumulation of IAA in cells. However, sulfonation of quercetin leads to the loss of its functions, thereby abolishing the inhibition of auxin efflux and stimulating the polar auxin transport (Faulkner and Rubery 1992). Furthermore, sulfonation is involved in the biosynthesis of phytohormones phytosulfokines, sulfonated tetra- and pentapeptides, which participate in the regulation of physiological processes, including induction of somatic embryogenesis, cell growth and cell development, regulation of responses to biotic/abiotic stress, and enhancement of root growth and nodulation in legumes (Di, et al. 2022; Li, et al. 2024; Matsubayashi and Sakagami 1996; Shen and Diener 2013). Phytosulfokines are biosynthesized in higher plants from polypeptides which are first sulfonated on the hydroxy groups of Tyr in the *cis*-Golgi apparatus by tyrosylprotein sulfotransferases (TPSTs) using 3'-phosphoadenosine-5'-phosphosulfate (PAPS) as a universal donor (Li, et al. 2024). Subsequently, sulfonated protein is cleaved into active sulfonated tetra- and pentapeptides (Li, et al. 2024). The biosynthesis of all other sulfonated metabolites is mediated by sulfotransferases (SOTs), which are typically present in the cytosol and, like TPSTs, can only use PAPS as a sulfate donor (Hirschmann, et al. 2014). In *Arabidopsis thaliana*, the *AtSOTs* family consists of 22 proteins, several of which have already been annotated. For example, *At*SOT15 is specific for the hydroxy derivatives of jasmonic acid (JA), which is involved in plant defense against mechanical damage caused by herbivores (Fernández-Milmanda, et al. 2020; Gidda, et al. 2003; Hirschmann, et al. 2014). The inactivation of hydroxy-JA by sulfonation leading to the reduction in JA signaling is specifically induced during the development of shade avoidance syndrome (Fernández-Milmanda, et al. 2020; Gidda, et al. 2003). Specifically, 12-hydroxyjasmonic acid is sulfonated to 12-(sulfooxy)jasmonic acid (HSO_4_-JA), being mediated by *At*SOT15, which is expressed in response to the decrease in the R:FR (red:far red) light ratio detected by phytochrome B. Thus, JA sulfonation results in defense attenuation at the expense of growth (Fernández-Milmanda, et al. 2020; Gidda, et al. 2003). Other annotated phytohormone-specific *At*SOTs include *At*SOT12, which is specific for salicylic acid (SA) and brassinosteroids, and *At*SOT10, which is specific only for brassinosteroids (Baek, et al. 2010; Lacomme and Roby 1996; Marsolais, et al. 2007). However, these sulfonated phytohormones have never been detected in plants (Hirschmann, et al. 2014).

In this study, a semi-targeted UHPLC–QqTOF-MS method was applied to 23 species to search for all putatively sulfonated metabolites. Among these, biologically relevant compounds were searched by interpreting the recorded MS/MS spectra. The main discovery was the identification of an unknown sulfonated auxin derivative in *Urtica dioica*. This novel metabolite was subsequently synthesized and partially characterized in terms of its biosynthesis and biological activity.

## Materials and methods

### Plant material

Seeds of stinging nettle (*Urtica dioica* L.) were purchased from SemenaOnline, s.r.o. (Jeneč, Czech Republic). Seeds of the DR5::GUS reporter line of *Arabidopsis thaliana* were kindly provided by Prof. Guilfoyle (University of Missouri, Columbia, USA). Seeds of *A. thaliana* (ecotype Col-0) used in the phenotyping study were acquired from the Salk Institute Genomic Analysis Laboratory (www.signal.salk.edu/cgi-bin/tdnaexpress). The initial screening was performed on freeze-dried plant and algae from the databases kept at the Department of Experimental Biology (Palacky University Olomouc), and Microalgae and Zygnematophyceae Collection Hamburg (Universität Hamburg); a complete list of species is given in supplementary materials (Tables S1 and S2).

### Growth conditions of *Urtica* and treatment with ^34^S-ammonium sulfate

*Urtica dioica* seeds were germinated on cellulose fiber in closed Petri dishes (5 cm Ø) with 3 mL of tap water in controlled conditions (23 °C; 16-/8 h light/dark). Seedlings from one Petri dish were used as one biological replicate. After 12 days of germination, water solution of isotopically labeled ^34^S-ammonium sulfate (≥ 98 atom % ^34^S; Merck, Darmstadt, Germany) was added to *Urtica* seedlings to final concentrations of 10 and 100 mM. Three biological replicates were prepared (n = 3). After 10 days of treatment, seedlings were collected, freeze-dried in a Gregor L10-55 lyophilizer (Gregor Instruments s.r.o, Czech Republic), homogenized, extracted and analyzed by a targeted UHPLC–MS/MS method.

### Treatment of *Urtica* by different light conditions

*Urtica dioica* seeds were sown in soil in square plastic pots (6 × 6 × 6 cm) in controlled conditions (23 °C, 16-/8-h light/dark) for 21 days. There were six plants in each pot, representing one biological replicate. The 21-day-old *Urtica* plants were exposed to continuous blue (B) light, continuous red (R) light, white (W) light in a 16-/8-h light/dark regime (light intensity 100 μmol m^−2^ s^−1^), or kept in the dark, which served as a control (Ctrl), for 7 days. The blue light was provided by fluorescent tubes Philips TLD-36W/18-Blue (Philips, USA) with maximum illumination 10 μmol m^−2^ s^−1^ at 440 nm. Fluorescent tubes Philips TLD-36 W/15-Red (Philips, USA) with maximum illumination 10 μmol m^−2^ s^−1^ at 660 nm were used as a source of red light. Samples were prepared in 3 biological replicates (*n* = 3). The 28 day-old plants were harvested, freeze-dried in a Gregor L10-55 lyophilizer (Gregor Instruments s.r.o), and homogenized in a coffee mill.

### Extraction

The extraction method has been described previously (Rárová, et al. 2019; Supikova, et al. 2022). Briefly, 20 mg of dry weight (DW) material was extracted with methanol (hypergrade for UHPLC, LiChrosolv, Merck, Darmstadt, Germany) containing internal standard of deuterium-labeled 4-hydroxybenzoic acid (2,3,5,6-^2^H_4_) purchased from Cambridge Isotope Laboratories Inc. (Tewksbury, USA). Extracts were evaporated under nitrogen, redissolved in 200 μL of 20% MeOH and analyzed by LC–MS methods.

### UHPLC–QqTOF-MS analysis and MS data processing

Details of the analysis can be found in our previous publications (Rárová, et al. 2019; Supikova, et al. 2022). Briefly, ultra-high-performance liquid chromatography with quadrupole time-of-flight mass spectrometry (UHPLC–QqTOF-MS) and with electrospray (ESI) operating in negative ion mode (ESI-) were employed for a semi-targeted analysis. Leucine-enkephalin served as a lock mass (concentration of 5 ng/μL). Mobile phases A (acetonitrile from LiChrosolv, Merck, Darmstadt, Germany) and B (5 mM formic acid from Merck) were mixed in a gradient elution. Deionized water was prepared using the Simplicity 185 system (Millipore Corp., Billerica, USA). Samples were separated on an ACQUITY UPLC® BEH C18 column (150 × 2.1 mm, 1.7 μm, Waters). Data were evaluated in MassLynx ™ software (ver. 4.0, Waters).

The raw MS data recorded in data-dependent acquisition mode were processed using in-house Matlab® algorithms to extract list of features and their MS^2^ spectra. This list was further reduced by removing features with peak area < 500. To detect putative sulfometabolites, the neutral loss of SO_3_ (Δ *m/z* 79.9568) was searched in the MS^2^ (Tab. S1). Additionally, *m/z* 116, 128 and 130 (for indole derivatives), *m/z* 93, 109 and 135 (for hydroxybenzoates) and *m/z* 134 (for adenine derivatives) were searched in the MS^2^. The detected sulfometabolites were putatively annotated/identified using MS databases, including Metlin (https://metlin.scripps.edu/), MassBank (https://massbank.jp/) and our local library. Authentic standards of 4-(sulfooxy)phenylacetic acid (SPAA), 4-(sulfooxy)benzoic acid, zosteric acid, and ferulic acid 4-sulfate were synthesized in our previous study (Supikova, et al. 2022). To identify these metabolites in the extracts, the RTs and MS^2^ spectra of the standards were compared with those of the metabolites from the samples. Putatively identified metabolites were elucidated using elemental compositions and MS^2^ spectra and included *N*-sulfoindole-3-acetic acid (SIAA), 4-(sulfooxy)phenyllactic acid (SPLA), *N*-sulfoindole-3-lactic acid (SILA), isoferulic acid 3-sulfate, HSO_4_-JA and HSO_4_-dihydro-JA. In addition, the pseudo-MS^3^ spectra of SIAA, SPLA and SILA corresponded to the MS^2^ spectra of IAA, 4-hydroxyphenyllactic acid (HPLA) and indole-3-lactic acid (ILA) (purchased from Merck). The standard of SIAA was synthesized for its complete identification.

### Quantification of SIAA

SIAA was quantified in *Urtica* plants treated with different light conditions using the UHPLC–QqTOF-MS analysis. The concentration of SIAA was determined in MassLynx™ software (Waters, Milford, USA) using an internal standard method and a regression calibration, which was calculated over 5 orders of magnitude (1·10^–8^ to 1·10^–4^ M solutions of standards). The limit of detection (LOD), calculated from a signal-to-noise (S/N) ratio of 3:1, was 289 fmol/injection, or 2.89 pmol/g DW.

### Targeted UHPLC–MS/MS method and incorporation of ^34^S-sulfate

The UHPLC conditions were the same as for the UHPLC–QqTOF-MS method. Micromass Quattro microTM API benchtop Z-spray ionization (Waters) was used for MS. Ionization was performed in ESI- mode. The following settings were used: source temperature 120 °C, desolvation temperature 350 °C, desolvation gas flow 500 L/h, cone voltage 20 V, capillary voltage 2 kV, and collision energy 25 eV. The analyses in multiple reaction monitoring (MRM) mode included (i) the targeted analysis of SIAA in all species from Tab. S2; (ii) the analysis of *Urtica* seedlings treated with ^34^S-sulfate. The LOD of SIAA was 24.4 fmol/injection, i.e. 244 fmol/g DW, calculated from calibration solutions of 1·10^–9^—1·10^–5^ M using the S/N ratio (> 3). Quantification transitions were 256 > 130 for ^34^S-SIAA (heavy isotope-containing) and 254 > 130 for ^32^S-SIAA (unlabeled). Confirmation transitions were 256 > 176 and 256 > 212 for ^34^S-SIAA, and 254 > 174 and 254 > 210 for ^32^S-SIAA. The range of masses scanned in MS^1^ and MS^2^ was 40–2048 Da. Data were processed and evaluated using MassLynx™ (ver. 4.0) software.

The ^34^S incorporation rate into SIAA in percentage was calculated by substituting the peak area of *m/z* 256 > 130 (^34^S-SIAA) and *m/z* 254 > 130 (^32^S-SIAA) determined in the control (ctrl) and ^34^S-sulfate-treated samples (treat) into the following equation:

$$Incorporation \,(\% ) = 100\left[ {\tfrac{{Area_{{34_{{S\left( {treat} \right)}} }} - Area_{{34_{{S\left( {ctrl} \right)}} }} }}{{Area_{{32_{{S\left( {treat} \right)}} }} + Area_{{34_{{S\left( {treat} \right)}} }} - Area_{{34_{{S\left( {ctrl} \right)}} }} }}} \right]$$

### Quantification of auxin metabolites

The determination of endogenous auxin metabolites followed the protocol described by Pencík, et al. (2018). In summary, approximately 1 mg of freeze-dried *Urtica* tissue was extracted with 1 mL of cold 50 mmol/L phosphate buffer (pH 7.0) containing 0.1% sodium diethyldithiocarbamate and a mixture of stable isotope-labeled internal standards. A 200 µl portion of the extract was acidified to pH 2.7 with HCl and subjected to in-tip micro solid phase extraction (in-tip µSPE). Another 200 µL portion was derivatized with cysteamine, acidified to pH 2.7 with HCl, and purified using in-tip µSPE to determine IPyA. After elution, the samples were evaporated under reduced pressure, reconstituted in 10% aqueous MeOH, and analyzed using HPLC system 1260 Infinity II (Agilent Technologies, USA) equipped with a Kinetex C18 column (50 mm × 2.1 mm, 1.7 µm; Phenomenex, Torrance, USA) and coupled to 6495 Triple Quad detector (Agilent Technologies, Santa Clara, USA).

### Synthesis of 2-(1-sulfo-1H-indol-3-yl)acetic acid

2-(1*H*-Indol-3-yl)acetic acid (175 mg, 1 mmol) was dissolved in pyridine (0.6 mL) to which sulfur trioxide-pyridine complex (159 mg, 1 mmol) was added. The resulting mixture was refluxed for 4 h. Subsequently, the reaction mixture was cooled down, diluted with water (10 mL), and extracted with diethyl ether (2 × 15 mL). The organic phase was discarded and the aqueous phase was evaporated to dryness. The resulting residue was purified by preparative thin layer chromatography (EtOAc/EtOH 4/1) and column chromatography (EtOAc/EtOH 9/1) to give 2-(1-sulfo-1*H*-indol-3-yl)acetic acid (74 mg, 29%) as a light pink solid. R_*f*_ = 0.13 (EtOAc/ MeOH/acetic acid 90/10/1). The NMR spectra are available as a supplementary file (Fig. S1).

^1^H NMR (500 MHz, DMSO-*d*_6_): δ 3.61 (2H, s, CH_2_), 7.03 (1H, t, *J* = 7.3 Hz, CH), 7.12 (1H, t, *J* = 7.4 Hz, CH), 7.37 (1H, s, CH), 7.44 (1H, d, *J* = 7.6 Hz, CH), 7.78 (1H, d, *J* = 8.3 Hz, CH), 12.15 – 12.35 (1H, br s, COOH). ^13^C NMR (125 MHz, DMSO-*d*_6_): δ 30.9 (CH_2_), 107.6 (C), 113.6 (CH), 118.5 (CH), 119.5 (CH), 121.6 (CH), 126.0 (CH), 128.5 (C), 134.7 (C), 173.2 (C).

### NMR

^1^H (500 MHz) and ^13^C (125 MHz) NMR spectra were recorded in DMSO-*d*_6_ at room temperature on a Jeol ECA-500 spectrometer equipped with a 5 mm Royal probe. Chemical shifts were calibrated to the residual solvent peak (*δ* = 2.50 ppm for ^1^H and *δ* = 39.50 for ^13^C). Signal coupling patterns are represented as singlet (s), broad singlet (br s), doublet (d), triplet (t), and multiplet (m). Coupling constants are reported in Hz.

### DR5::GUS activity of SIAA

A DR5::GUS reporter line of *Arabidopsis thaliana* was used to define a possible agonist or antagonist effect of SIAA on IAA. The DR5::GUS seeds were placed in 96-well microplates containing half-strength MS medium and stratified at 4 °C in the dark for 4 days. The microplates were then transferred to a phytotron under controlled conditions (75 rpm, 21 °C, 16-/8-h light/dark, light intensity 100 μmol m^−2^ s^−1^) for 7 days, after which the different concentrations of SIAA were added. In addition, 5 μM IAA dissolved in 0.1% acetic acid was prepared and added to a different set of *A. thaliana* seedlings to obtain a positive control. Negative controls were achieved by applying a half-strength MS medium with or without 0.1% acetic acid. After 17 h of incubation, the medium was discarded, and the seedlings were treated directly with 150 μL lysis buffer (50 mM sodium phosphate, pH 7.0, 10 mM EDTA, 0.1% Triton X-100) containing 1 mM of 4-methyl-umbelliferyl glucuronide, and incubated at 37 °C for 90 min. At the end of the incubation period, 50 μL of 1 M Na_2_CO_3_ (stop solution) was added to each well, and the fluorescence due to 4-methylumbelliferone formation was measured in a microplate reader (excitation/emission wavelengths 365/460 nm).

### Phenotype analysis of *Arabidopsis thaliana* treated with SIAA

Seeds of *A. thaliana* (Col-0) were surface sterilized with 70% EtOH according to the protocol described by De Diego, et al. (2017). Seeds were homogeneously distributed on a filter paper moistened with water. After 4 min, the filter paper with the seeds was transferred to a square plate (120 × 120 mm) containing half-strength solid MS medium (Phytotechlab M519) supplemented with SIAA, IAA, or without compound (Ugena, et al. 2018). After sowing, the square plates were kept in the dark at 4 °C for 4 days, followed by another 4 days in the vertical position in the growth chamber under controlled conditions (16-/8-h light/dark, 22 °C, light intensity 120 μmol m^−2^ s^−1^ and relative humidity of 60%). The seedlings were then transferred to 48-well plates with 1 × MS medium (pH 5.7, 0.6% Phytagel). The plates were placed in the OloPhen platform, which uses the PlantScreenTM XYZ system in a growth chamber with a controlled environment (22 °C/20 °C regime in a 16-/8-h light/dark cycle, light intensity 120 μmol m^−2^ s^−1^ and a relative humidity of 60%) (De Diego, et al. 2017). The plates were photographed twice daily for 7 days using a red–green–blue camera (Ugena, et al. 2018). Features extracted from the image analysis were, e.g. the early seedling establishment (the size of the seedlings on the 1st day) defining the effect of the compound pretreatment, and the final area and perimeter of the seedlings on the last day. Further details can be found in Ugena, et al. (2018).

### Statistical analysis

The significance was assessed using unpaired two-tailed Student's *t* test at *P < 0.05, **P < 0.01 and ***P < 0.001 in Excel (Microsoft) The hierarchical cluster analysis of metabolites was performed using Ward's method (PAST software 4.03).

## Results

### Identification of sulfonated auxin in stinging nettle (*Urtica dioica*)

A semi-targeted metabolomic profiling approach addressed the knowledge gap regarding unknown sulfated metabolites and their precursors. A selection of 23 plant, algal and fungal species (Tab. S1), including different tissues/organs such as leaves and fruits, were analyzed by the UHPLC–QqTOF-MS analysis, with MS operating in negative ion mode (ESI-), which allows easy ionization of acidic sulfonated molecules. Sulfometabolites were putatively identified based on the presence of a neutral loss of sulfur trioxide, as indicated by the *m/z* difference of 79.9568 Da in the MS^2^ spectra.

**Fig. 1:**
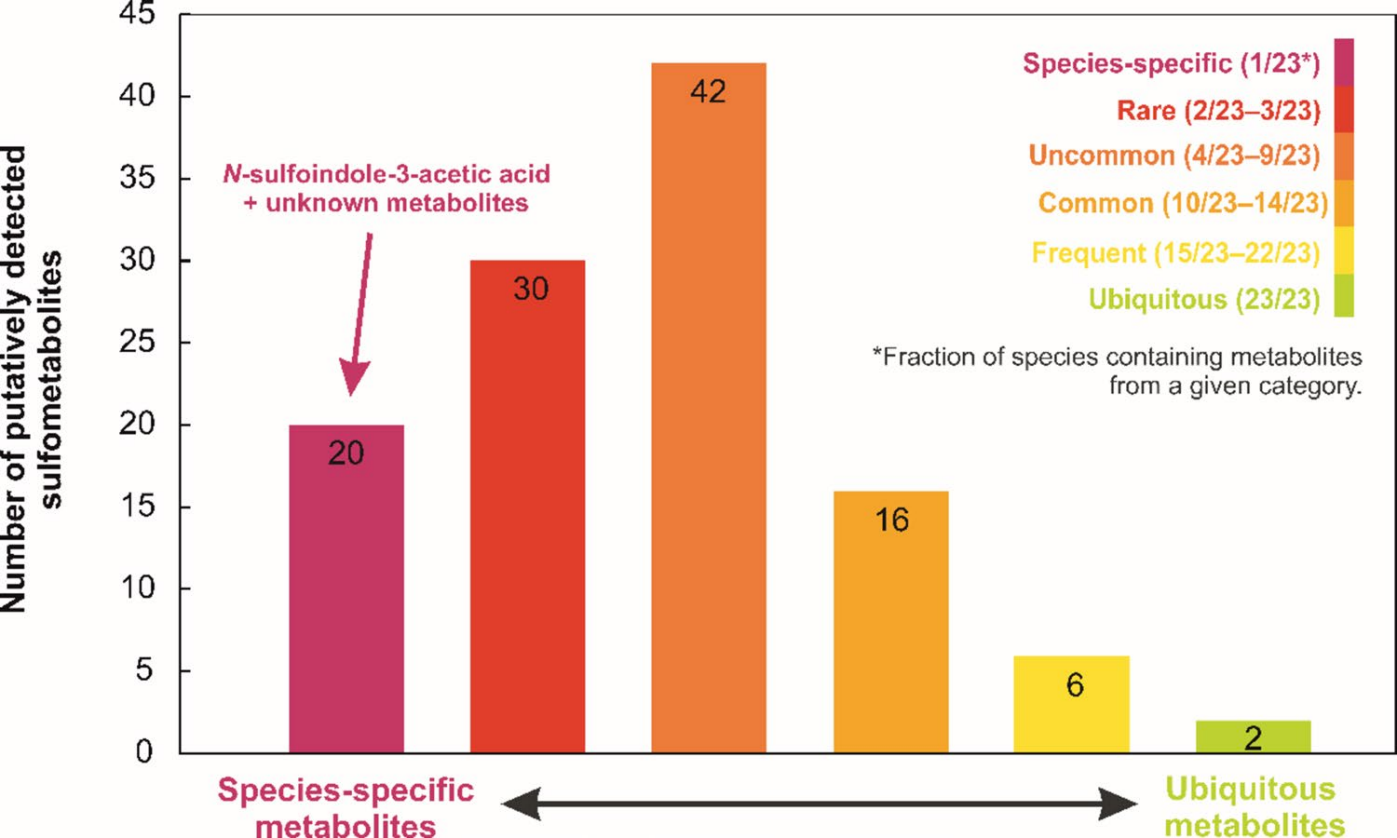
A semi-targeted UHPLC–QqTOF-MS analysis of 23 plant/algae/fungi species resulted in the detection of 116 putative sulfometabolites, which were identified based on the detection of the neutral loss of SO_3_ (Δ*m/z* 79.9568 Da) in the MS^2^ spectra. These sulfometabolites were classified into six categories based on the number of species in which they were detected. The species-specific metabolites detected in only 1/23 species are indicated in magenta. The rare metabolites occurring in 2/23–3/23 species are indicated in red. The uncommon metabolites occurring in 4/23–9/23 species and common metabolites occurring in 10/23–14/23 species are indicated by orange colors. The frequent metabolites occurring in 15/23–22/23 species are indicated in yellow. The ubiquitous metabolites detected in all species are indicated in green (color figure online)

As a result, 116 putative sulfometabolites with predominantly unidentified structures were obtained and classified into six groups, as shown in the histogram (Fig. 1; Tab. S1), based on their distribution among the analyzed species. Given the considerable number of these metabolites, further structural characterization was not performed. This result is consistent with the assumption that plant sulfometabolites represent a large and heterogeneous group of metabolites that have not yet been fully characterized. Of the 116 sulfometabolites, 20 showed a species-specific distribution within the analyzed species, being present in only 1 out of 23 species (Fig. 1). 30 metabolites were present in only 2–3 out of the 23 species; therefore, they were classified as rare (Fig. 1). Other categories were also outlined, with each category defined by the fraction of species in which a particular metabolite was detected. These categories included uncommon, common and frequent metabolites, which were present in 4–9 out of 23 species, 10–14 out of 23 species and 15–22 out of 23 species, respectively (Fig. 1). Only 2 metabolites of unknown structure were detected in all 23 species and thus were classified as ubiquitous metabolites, which are presumably also widespread in nature (Fig. 1).

4 of the 116 putative sulfometabolites were identified: SPAA, 4-(sulfooxy)benzoic acid, zosteric acid and ferulic acid 4-sulfate. Identification of these metabolites was based on a comparison of their MS^2^ spectra and RTs with those of authentic standards. In addition, HSO_4_-JA, HSO_4_-dihydro-JA, and isoferulic acid 3-sulfate were putatively annotated based on their MS^2^ spectra (Tab. S1).

Among the remaining 109 putative sulfometabolites, we further searched for biologically relevant compounds, such as sulfonated phytohormones, growth regulators, and primary metabolites that have not yet been identified in plants. The search was based on the prediction of the elemental composition of molecular ions [M-H]^−^, e.g. sulfonated SA, *m/z* 137 + 80 (SO_3_); sulfonated brassinolide, *m/z* 479 + 80 (SO_3_); and sulfonated castasterone, *m/z* 463 + 80 (SO_3_). In addition, the neutral losses and fragments typical to cytokinins (e.g. adenine, *m/z* 134), auxins (e.g. indole, *m/z* 116, 128, and 130), phenolic compounds (e.g. hydroxybenzoate, *m/z* 93, 109, and 125), and other relevant metabolites were searched among the MS^2^ spectra. Matching candidate metabolites were further elucidated by comparing their pseudo-MS^3^ spectra, obtained by in-source desulfonation of molecular ions, with MS^2^ spectra of non-sulfonated analogs available in databases.

**Fig. 2:**
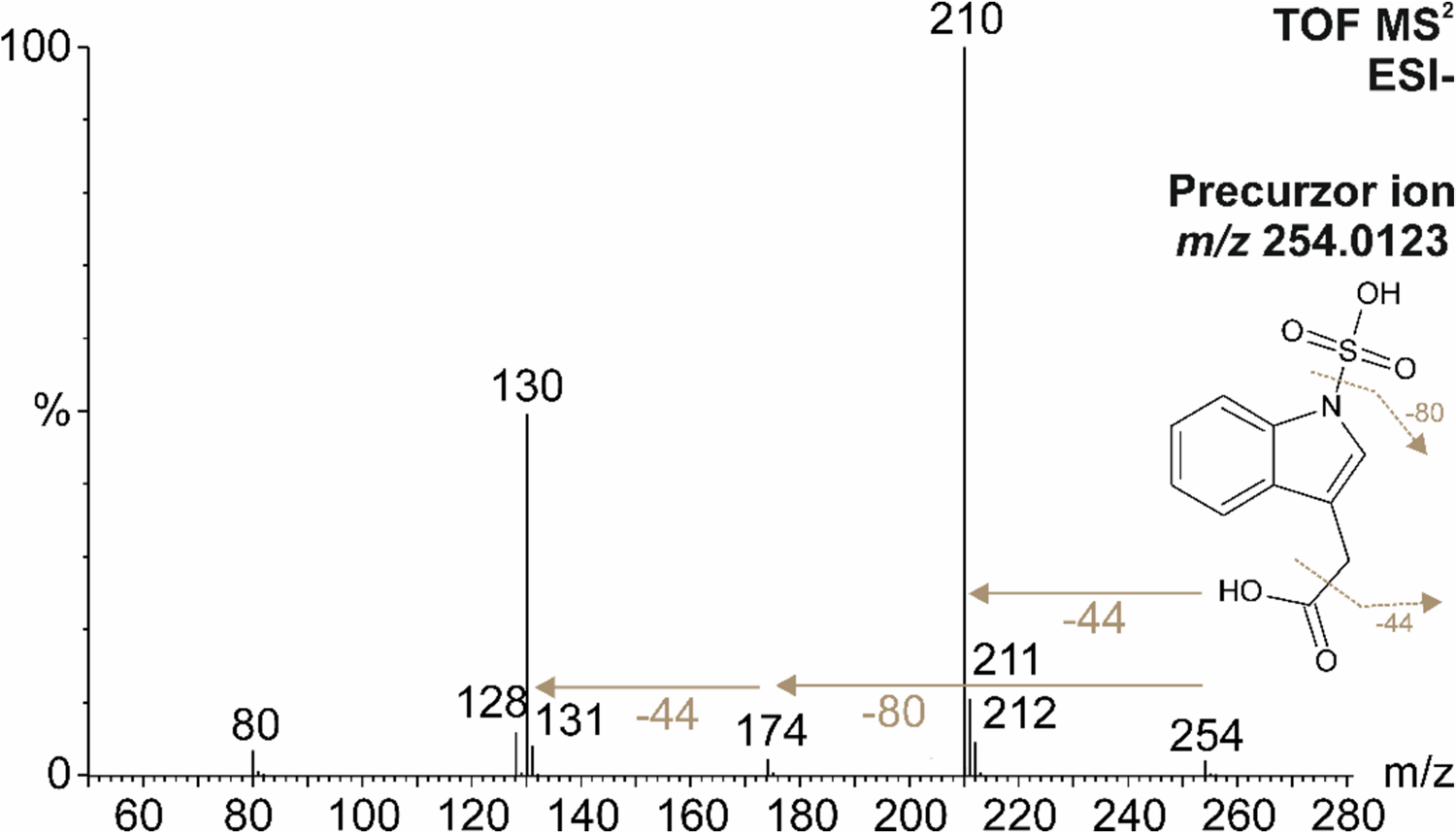
Fragmentation spectrum of N-sulfoindole-3-acetic acid (SIAA) with m/z 254.0123 identified in stinging nettle (*Urtica dioica*) using UHPLC–QqTOF-MS analysis in negative ion mode (ESI-). The collision energy was -20 eV. The neutral losses of SO_3_ (−80) and CO_2_ (-44) are indicated by brown arrows (color figure online)

This approach led to the preliminary identification of a sulfonated IAA, a novel derivative of the ultimate auxin phytohormone. This metabolite was detected in stinging nettle (*Urtica dioica*) as a molecular ion of [M-H]^−^ (*m/z* 254.0123), corresponding to a molecular formula of C_10_H_9_NO_5_S (Δppm 0.4). Since sulfonated IAA and the corresponding SOT for auxin sulfonation have never been reported in plants, the structure was further elucidated manually. The methylindole substructure, which is typical for the MS^2^ spectrum of IAA and other indole derivatives, was distinguished by two characteristic fragments with *m/z* 128.0564 and 130.0668 in the MS^2^ spectrum (Fig. 2). Similar to the MS^2^ spectrum of IAA, SIAA also showed a neutral loss of carbon dioxide (CO_2_) from the carboxyl group, as indicated by fragments with *m/z* 130 (C_9_H_8_N) and *m/z* 210 (C_9_H_8_NO_3_S) (Fig. 2). The neutral loss of SO_3_, indicated by *m/z* 174 (C_10_H_8_NO_2_), was of low intensity compared to *O*-sulfonated compounds such as phenylsulfates, where the [M-SO_3_-H]^−^ ion is typically the most intense in MS^2^ (Supikova et al. 2022). Due to (i) the loss of CO_2_ from the carboxyl group and (ii) the almost non-existent metabolites containing a -C-SO_3_H group, the newly discovered metabolite was putatively annotated as *N*-sulfoindole -3-acetic acid (SIAA), with the sulfo group attached to the nitrogen in the indole ring.

**Fig. 3:**
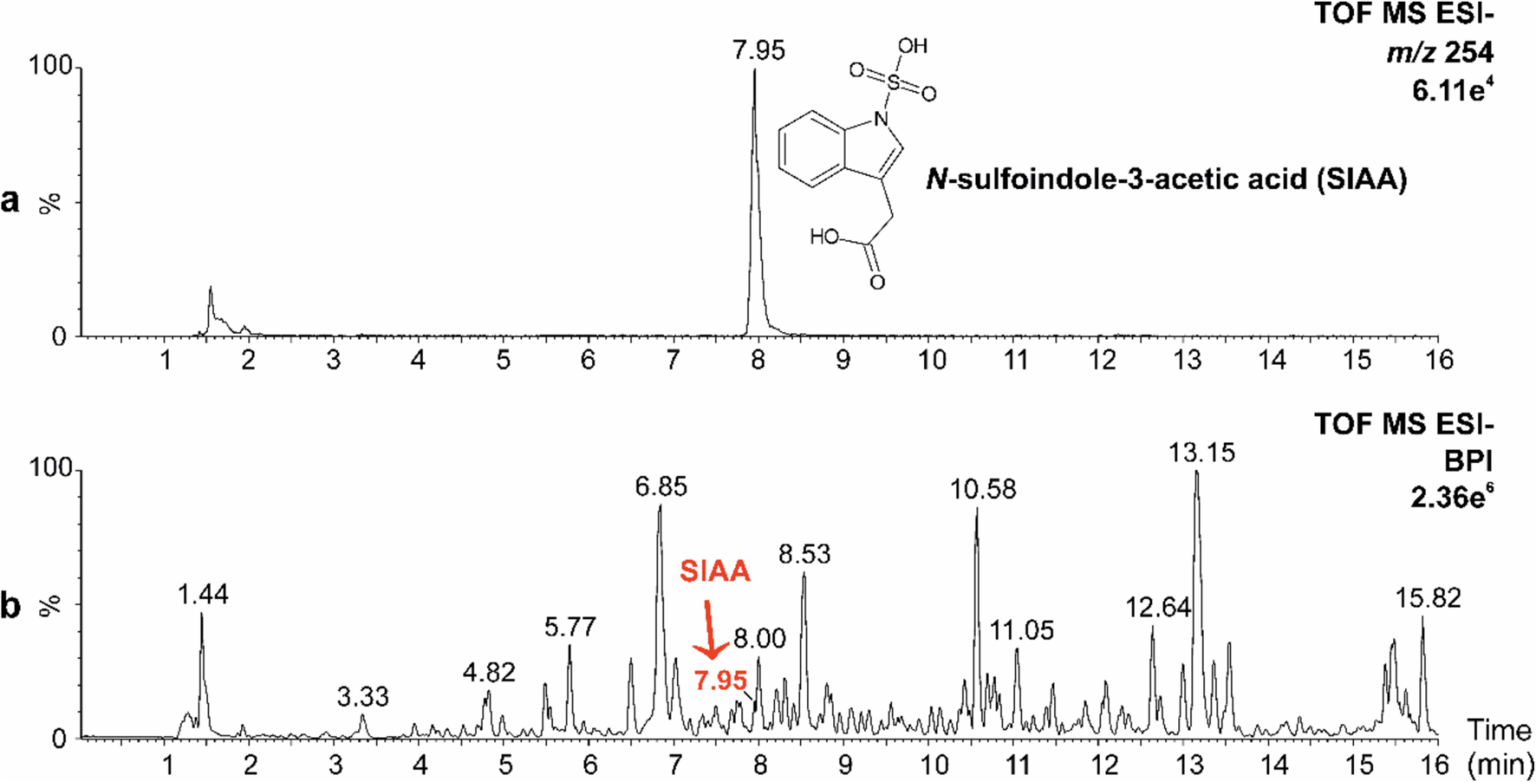
**a** Extracted ion chromatogram of *m/z* 254 showing *N*-sulfoindole-3-acetic acid (SIAA) at RT of 7.95 min in an extract of above-ground tissues of stinging nettle (*Urtica dioica*) which was analyzed by UHPLC–QqTOF-MS in negative ionization mode (ESI-). **b** Base peak ion (BPI) chromatogram of an extract of above-ground tissues of stinging nettle (*Urtica dioica*) with a peak of SIAA, marked with a red arrow, at RT of 7.95 min

To validate the predicted structural identification, we synthesized the standard of SIAA and compared its exact mass (*m/z* 254.0123), RT (7.96 min), and MS^2^ spectrum with those of the discovered metabolite (Figs. 2 and 3; Fig. S2 and S3) under the same experimental conditions. The RT and MS parameters of the standard matched those of the metabolite, thereby confirming the proposed structure of SIAA. The concentration of SIAA in *Urtica dioica* plants was determined by a targeted UHPLC–QqTOF-MS analysis using an internal standard method. The concentration of SIAA was 13.906 ± 9.603 nmol/g DW, i.e. 6.62 ± 1.18 μg/g DW, in *Urtica* plants grown in W light (Tab. S3). In addition, a more sensitive method using triple quadrupole MS operating in MRM mode had to be applied for the quantitative analysis of IAA and its derivatives. The resulting concentration of IAA was almost 30 times lower than that of SIAA, i.e. 482 ± 111 pmol/g DW (Tab. S3).

As SIAA was only identified in *Urtica* (Fig. 3b) during our preliminary search, a targeted approaches using either QqTOF-MS or triple quadrupole (QqQ) operating in MRM mode were applied to a broader range of species (Tab. S2). This extended analysis included *Urtica* seeds and key model organisms, including *Arabidopsis thaliana*, *Physcomitrium patens,* wheat (*Triticum aestivum*), barley (*Hordeum vulgare*), oat (*Avena sativa*), maize (*Zea mays*), and tomato (*Solanum lycopersicum*). However, SIAA was detected again only in *Urtica*, particularly in shoot/seedlings but not in the seeds. Thus, among the 73 analyzed species in total, SIAA appears to be species-specific (Fig. 1).

### Identification of additional auxin-related sulfonated metabolites

**Fig. 4:**
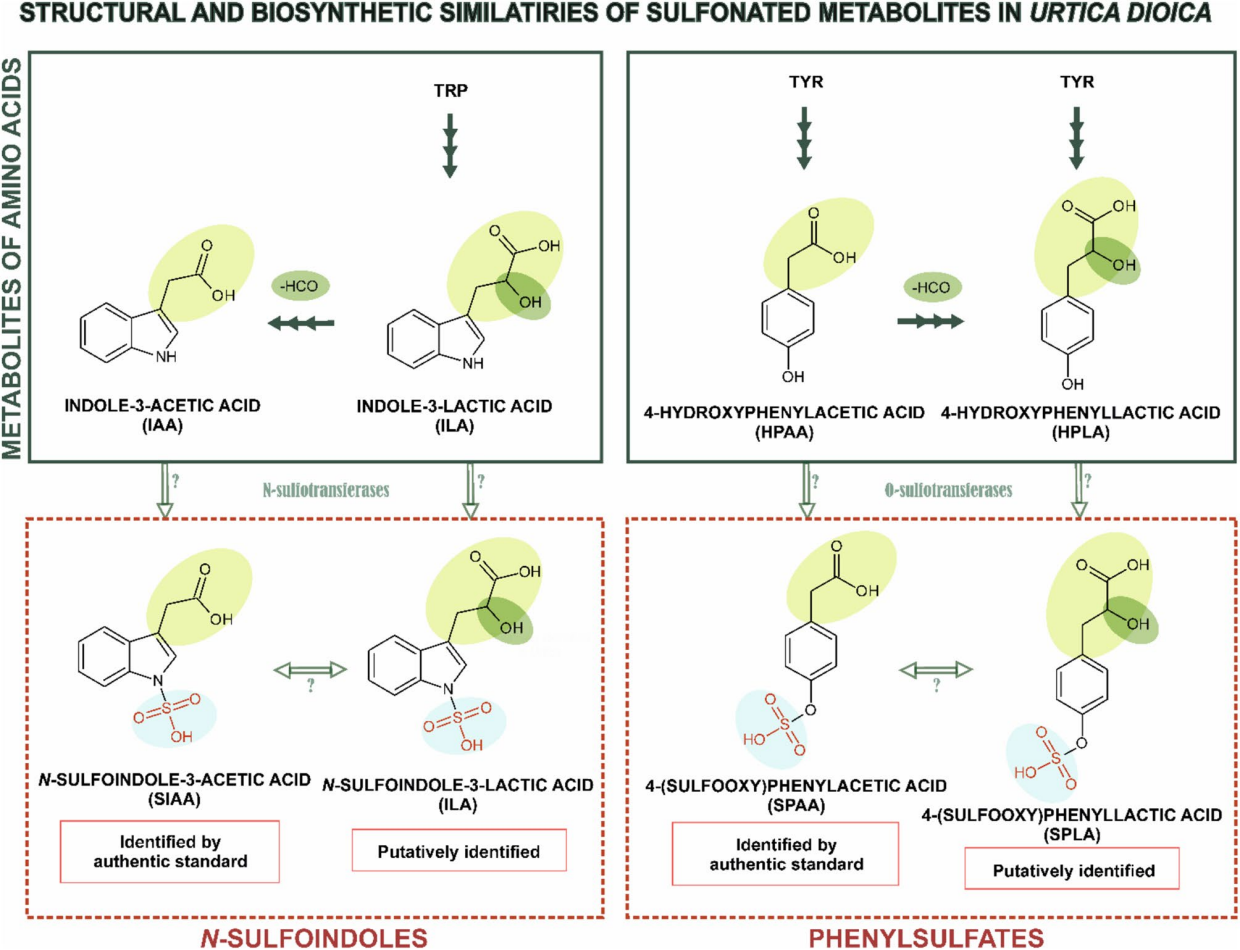
Structural and biosynthetic similarity of unknown sulfometabolites detected in *Urtica dioica* and their corresponding non-sulfonated forms. Proposed pathways are based on KEGG database (https://www.genome.jp/kegg/pathway.html). Sulfoindoles: *N*-sulfoindole-3-acetic acid (SIAA) and *N*-sulfoindole-3-lactic acid (SILA); phenylsulfates: 4-(sulfooxy)phenylacetic acid (SPAA) and 4-(sulfooxy)phenyllactic acid (SPLA)

Another *Urtica* metabolite, SILA, which is structurally related to SIAA (Fig. 4), was putatively identified as a molecular ion of [M-H]^−^ (*m/z* 284.0233; RT 6.78 min; C_11_H_10_O_6_S, Δppm 0.4) based on a characteristic neutral loss of SO_3_ in MS^2^, producing a fragment of *m/z* 204 whose pseudo-MS^3^ spectrum corresponded to that of ILA. SILA and SIAA are structurally similar to another pair of auxin-related sulfonated metabolites, SPAA and SPLA, both of which were identified in *Urtica* (Fig. 4). SPAA was identified using an authentic standard. SPLA was putatively identified as a molecular ion of [M-H]^−^ (*m/z* 260.9400; RT 3.96 min; C_9_H_10_O_7_S, Δppm 0.2) based on the neutral loss of SO_3_ in MS^2^, producing a fragment of *m/z* 181 whose pseudo-MS^3^ spectrum matched with that of HPLA. The structural and putative biosynthetic relationships between the identified *N*-sulfoindoles, phenylsulfates and their respective non-sulfonated forms are shown in Fig. 4. MS^2^ spectra of SILA and SPLA are provided in supplementary materials (Fig. S4).

### Sulfonated IAA has no auxin activity

**Fig. 5:**
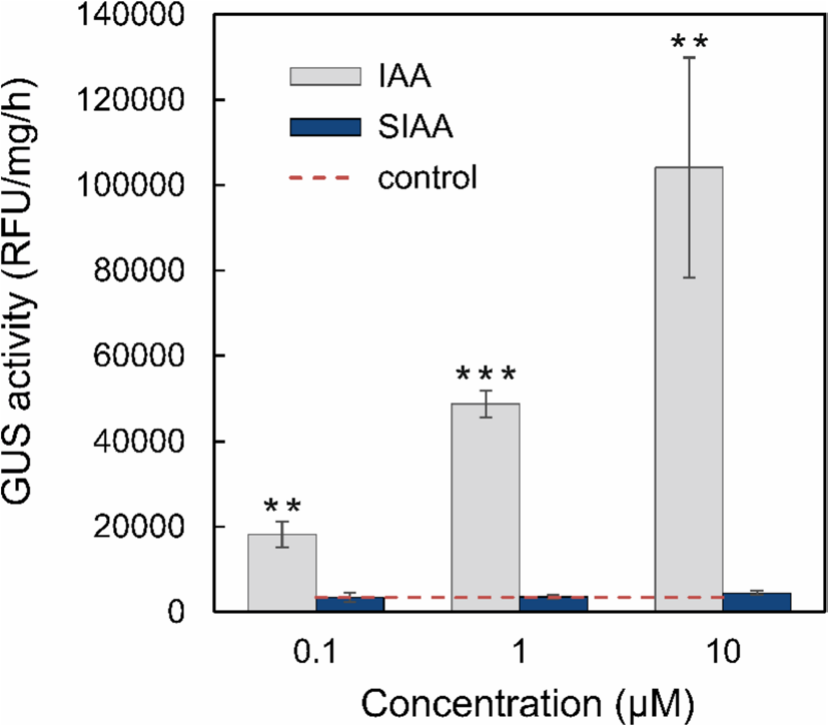
GUS activity of SIAA in DR5::GUS transformed *Arabidopsis thaliana* seedlings. The 7-day-old seedlings were treated with SIAA and IAA at concentrations of 0.1, 1 and 10 μM overnight (17 h). IAA was used as a positive control. Half-strength MS medium was used as a negative control. GUS activity was measured immediately after the treatment. Results are expressed as the mean (n=3). Error bars represent the standard deviation (SD). Unpaired two-tailed Student's *t* test was used to assess the significance (***P<0.001; **P<0.01)

The auxin activity of SIAA was investigated in *Arabidopsis thaliana* using a DR5::GUS reporter system, which allows quantitative assessment of an auxin response based on binding the ARF transcription factor to the auxin-responsive promoter DR5. The 7-day-old *Arabidopsis* seedlings were treated with SIAA and IAA, and GUS activity was analyzed in plant lysates the following day. The activity of 0.1 μM SIAA was only at the background level, whereas IAA, a positive control, induced intense GUS activity that was 5.4 times higher than that of SIAA (Fig. 5). A slight activation of DR5 by SIAA was detected at a concentration of 10 μM, being only 25% higher than the control, while 10 μM IAA induced more than a 30-fold increase in GUS activity (Fig. 5). Thus, the results show that sulfonation of IAA leads to the elimination of auxin activity.

### Incorporation of labeled ^34^S-sulfate into SIAA and related metabolites

Since *Urtica dioica* seeds, in contrast to adult plants and seedlings, do not contain detectable levels of SIAA, it seems obvious that SIAA is biosynthesized de novo after germination. A feeding experiment with isotopically labeled ^34^S-ammonium sulfate was proposed and performed on *Urtica* seedlings to confirm this hypothesis. The seedlings were treated with 10 mM and 100 mM ^34^S-ammonium sulfate for 10 days, after which they were harvested and analyzed by a targeted UHPLC–MS/MS method.

**Fig. 6:**
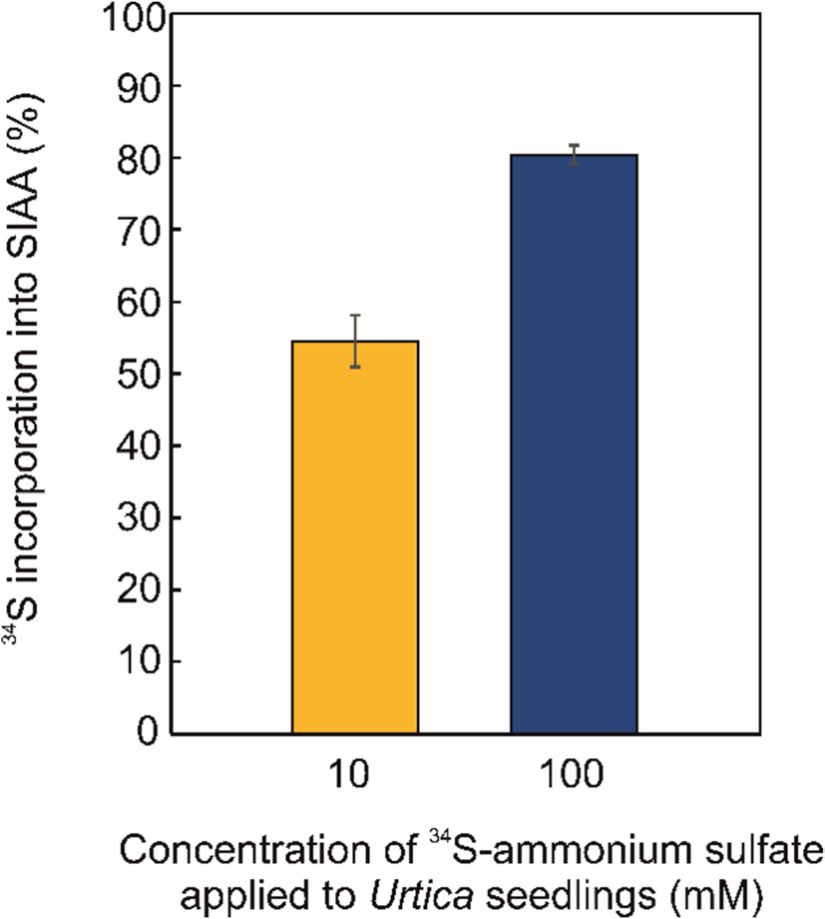
Percentage of ^34^S in SIAA in treated plants after the correction for naturally occurring heavy isotopes (^34^S, ^15^N, ^13^C, ^2^H), thus the incorporation rate in the control is 0%. *Urtica dioica* seedlings were treated with isotopically labeled ^34^S-ammonium sulfate for 10 days. A water solution of ^34^S-ammonium sulfate was added to the seedlings grown in Petri dishes to a final concentration of 10 and 100 mM. Peak areas of labeled ^34^S-SIAA and unlabeled ^32^S-SIAA were determined by UHPLC–MS/MS using MRM transitions of 256>130 (^34^S-SIAA) and 254>130 (^32^S-SIAA), respectively. Results are expressed as the mean (n=3). Error bars represent the SD

The incorporation of the exogenous ^34^S-sulfate into SIAA was determined from the ratio of ^34^S-SIAA (*m/z* 256 > 130) to ^32^S-SIAA (*m/z* 254 > 130) after correction to naturally occurring heavy isotopes (^34^S, ^15^N, ^13^C), which together accounted for 5.9 ± 0.3%. This result is consistent with the predicted natural abundance of these heavy isotopes of 5.62%. Thus, the intensity of ^34^S-SIAA (*m/z* 256 > 130) in the untreated control seedlings was 0% after correction. In the treated seedlings, 54.5 ± 3.6% and 80.3 ± 0.1% of the total SIAA contained ^34^S-sulfur originating from exogenously added 10 mM and 100 mM ^34^S-ammonium sulfate, respectively (Fig. 6). The results demonstrate that *Urtica* seedlings assimilate, metabolize and incorporate inorganic sulfate anion into SIAA, confirming its de novo biosynthesis. Furthermore, ^34^S incorporation into other auxin-related sulfonated metabolites was analyzed in treated *Urtica* seedlings. The incorporation of ^34^S was confirmed for SPLA and SILA, thereby providing evidence of their biosynthesis *in planta*. However, the incorporation of ^34^S-sulfate into SPAA was not confirmed.

### The effect of different light conditions on concentrations of SIAA and IAA derivatives

**Fig. 7:**
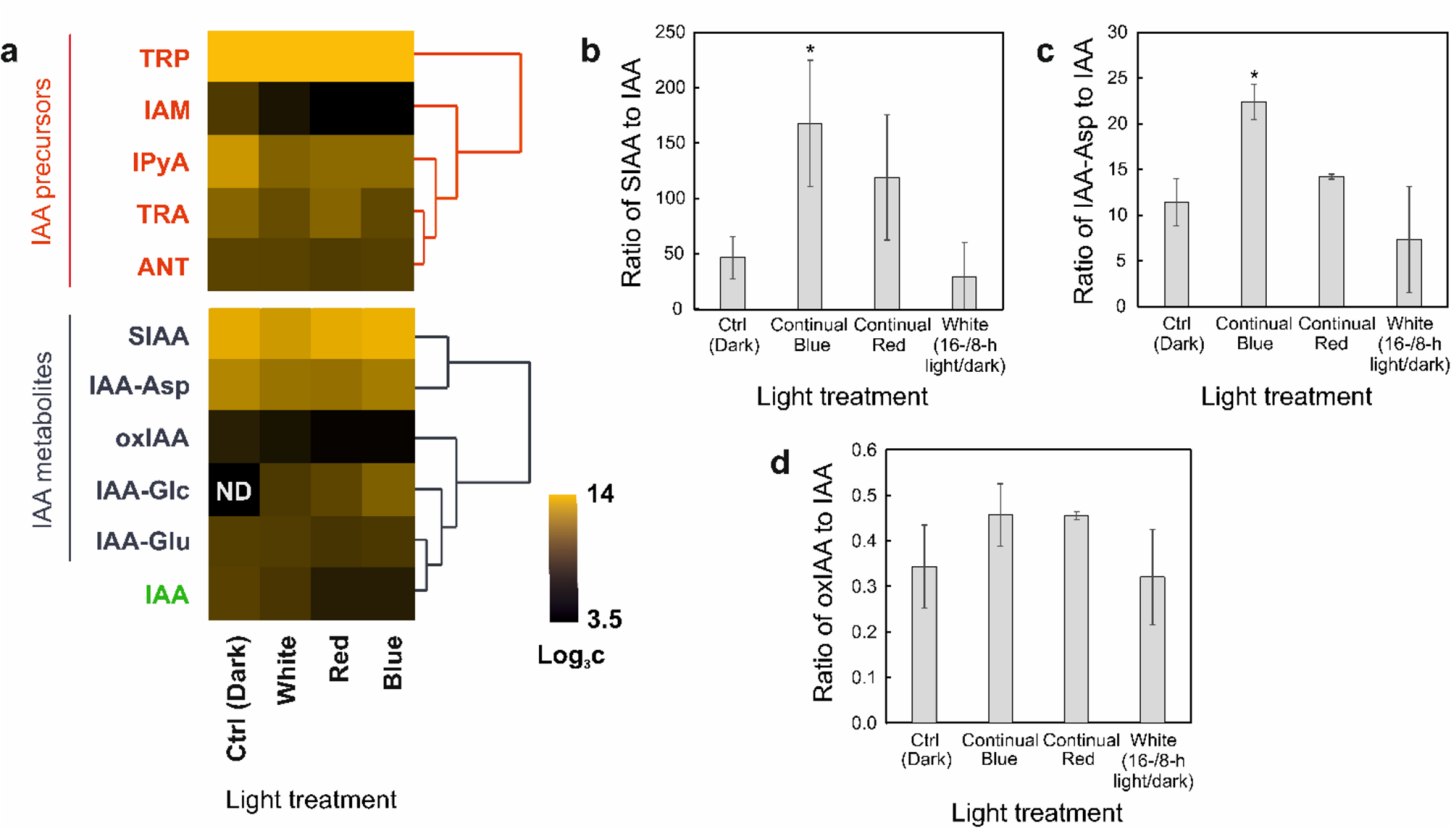
**a** Heat map of IAA metabolites and precursors detected in stinging nettle (*Urtica* *dioica*) grown under different light conditions, including continuous B and R light, W light (16-/8-h light/dark) and dark, which served as control (Ctrl). 21-day-old plants were transferred to growth chambers and analyzed by LC–MS/MS after 7 days of treatment. SIAA was quantified by UHPLC–QqTOF-MS analysis using an internal standard method. The other metabolites were quantified by LC–MS/MS according to the protocol described in Pencík, et al.^2018^. IAA-Glc was not detected (ND) in Ctrl. Results are expressed as means (n=3). Log_3_ was applied to the resulting concentrations (pmol/g DW). Metabolites were clustered based on log_3_ c data in Past software (ver. 4.03) according to Ward's method. The concentration ratios of **b **SIAA to IAA, **c **IAA-Asp to IAA and **d** oxIAA to IAA were determined in *Urtica* plants grown under different light conditions. Results are expressed as means (n=3). Error bars represent the SD. Unpaired two-tailed Student's* t* test was used to assess the significance (*P<0.05)

The effect of continuous B and R lights, W light (16-/8-h light/dark), and dark (Ctrl) on the levels of IAA and its metabolites and precursors was analyzed in *Urtica* plants. Under all light conditions, SIAA was detected as the most abundant auxin metabolite, except for a primary metabolite Trp (Fig. 7a; Tab. S3). In the control dark-developed plants, the concentration of SIAA was more than 40 times higher than that of IAA and IAA-Glu, more than 3.6 times higher than that of IAA-Asp, and more than 120 times higher than that of oxIAA, a degradation product of IAA (Fig. 7a–d; Tab. S3). IAA-Glc was only detectable under B, R, and W light conditions and was 20 to 32 times less abundant than SIAA (Fig. 7a; Tab. S3).

Since SIAA is likely biosynthetically related to IAA through one-step sulfonation, the effect of different light conditions on the levels of these two metabolites in particular was further investigated in *Urtica* plants. The SIAA/IAA ratio increased significantly in B light (3.5 times) and moderately in R light (2.5 times) compared with the control (Fig. 7b). In W light, the SIAA/IAA ratio showed a non-significant decrease (1.6 times) (Fig. 7b). Interestingly, in R light, the increase in SIAA/IAA was primarily due to reduced IAA levels, whereas in B light, the increase was further enhanced by the increased concentration of SIAA itself (Fig. 7a, b; Tab. S3). A similar ratio profile to that of SIAA was observed for IAA-Asp, a storage metabolite, where the Asp-IAA/IAA ratio increased significantly in B light (Fig. 7c). In contrast, the oxIAA/IAA ratio, which reflects the degradation of IAA, was not significantly altered by the applied light conditions and showed > 100 times lower values than the SIAA/IAA ratio (Fig. 7d).

### Sulfonation of IAA is stable *in planta*

We have shown here that sulfonation of IAA leads to the inactivation of auxin activity (Fig. 5). To build on this result, we tested the biological stability of SIAA and compared its potential auxin effect with IAA in the *Arabidopsis* model system. Although SIAA was not identified in *Arabidopsis* species in this study, we used the previously developed method based on the analysis of *Arabidopsis* shoot growth dynamics (De Diego, et al. 2017; Ugena, et al. 2018) because of its high sensitivity and predictive value to exogenously applied compounds (Ugena, et al. 2018). *Arabidopsis* seeds were primed with SIAA or IAA at 1 and 0.1 μM concentrations for 8 days (4 in dark and 4 in light for germination). The primed seedlings were transferred to a fresh medium for further growth (0–220 h) during which phenotypic parameters were quantitatively assessed. Rosette size was calculated from the number of green pixels in the photographed images and then averaged. Relative rosette size was expressed as T_n_/T_o_ – rosette size at time n (T_n_) divided by rosette size at time 0 (T_0_).

**Fig. 8:**
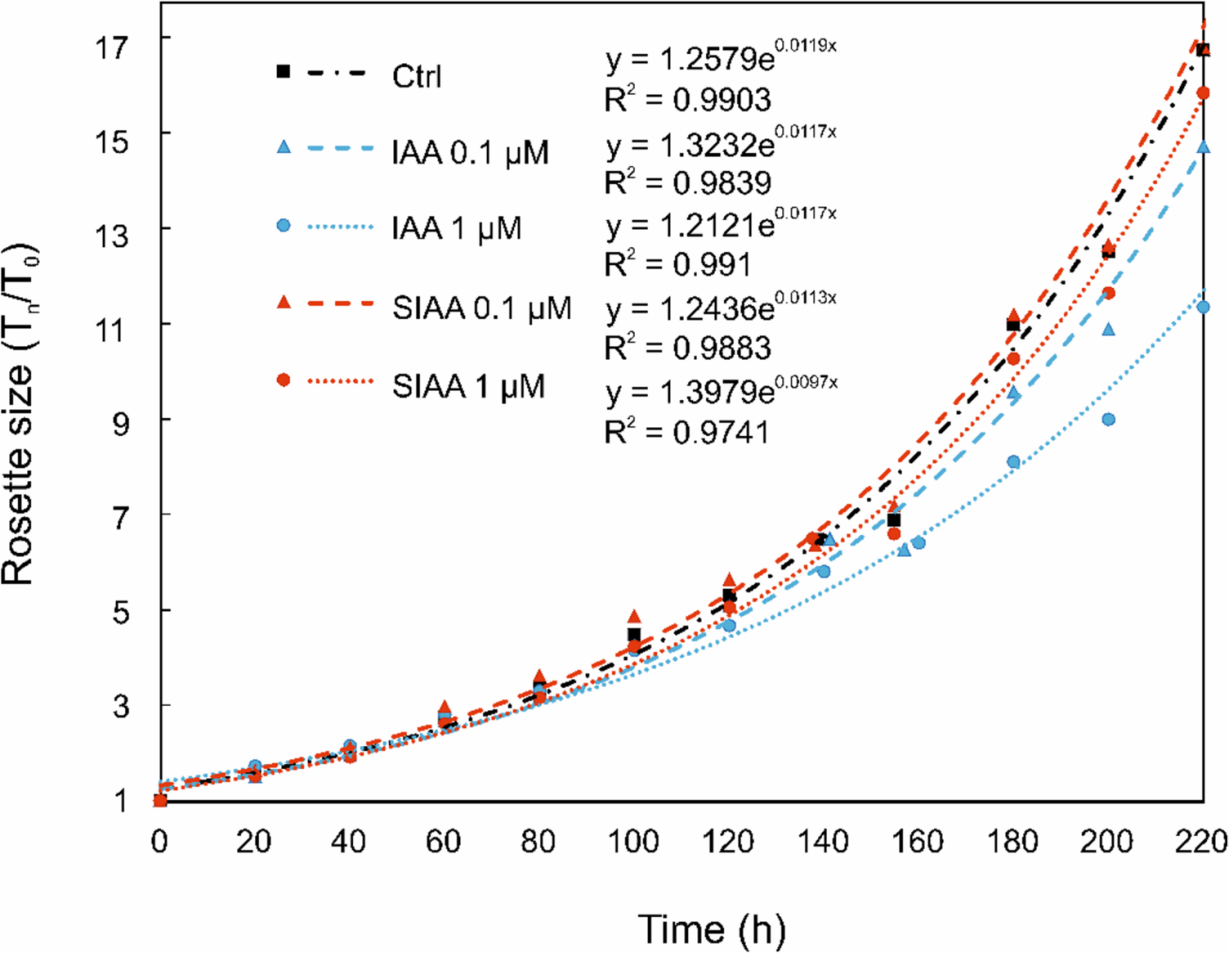
The effect of SIAA and IAA on *Arabidopsis *rosette size was evaluated in a previously developed phenotype analysis. (De Diego, et al. 2017; Ugena, et al. 2018) *Arabidopsis thaliana* seedlings were germinated for 8 days on half-strength MS medium supplemented with 0.1 and 1 μM compounds (SIAA, IAA) and no compounds as control (Ctrl). Plants were subsequently transferred to 1× MS medium to record rosette size during the growth for 220 h in 20 h intervals. Rosette size was expressed in relative units, corresponding to the ratio of the rosette size at time n (T_n_) to the rosette size at time 0 (T_0_)

In contrast to IAA, SIAA had no significant effect on the rosette size of *Arabidopsis* (Fig. 8). At a concentration of 0.1 μM, *Arabidopsis* rosettes treated with SIAA were larger than the untreated control plants. At concentrations of 1 μM, SIAA slightly inhibited rosette growth, but still less than 0.1 μM IAA, whereas 1 μM IAA inhibited rosette growth the most. The relative rosette size, T_n_/T_o_, of these treated plants was approximately 1/3 less than that of the control at the endpoint of 220 h. These results are consistent with the results of the GUS activity assay (Fig. 5), although the seedlings in the phenotype analysis were exposed to SIAA for a longer time (8 days) compared to the overnight exposure in the GUS activity assay. In addition, GUS activity was measured immediately after treatment. Hence, the SIAA seems to be a biologically stable inactive form without auxin effects in a plant system.

## Discussion

### Semi-targeted analysis of sulfometabolites

Although sulfonation is less explored compared to, for example, glycosylation, methylation, acetylation and amino acid conjugation, it results in a considerable number of sulfometabolites such as glucosinolates, sulfonated flavonoids, phenylsulfates, phytosulfokines and HSO_4_-JA (Cheynier, et al. 2013; Koprivova and Kopriva 2016; Ludwig-Muller 2011; Miersch, et al. 2008). In this study, sulfometabolites were putatively annotated in LC–MS/MS metabolomic data of 23 plant, algal and fungal species (Tab. S1) based on the detection of neutral loss of SO_3_. Putative sulfometabolites were categorized into six groups, reflecting their supposed natural distribution (Fig. 1). The majority of 116 metabolites were only present in a small number of species and were therefore classified as uncommon, rare or even species-specific (Fig. 1). This might reflect the fact that most detected compounds were derived from specialized secondary metabolites, that are typical for a particular or limited number of plant species (e.g. paclitaxel from *Taxus brevifolia*) (Wani, et al. 1971). For example, the sulfonated secondary metabolites taxifolin-7-sulfate, dihydrokaempferol-7-sulfate and eriodictyol-7-sulfate were only identified in *Salix* × *alberti* but not in other *Salix* spp. (Noleto-Dias, et al. 2020).

### N-sulfonated IAA identified in *Urtica* as a novel auxin modification

In this study, an unknown auxin metabolite substituted at indole nitrogen was discovered and structurally identified as *N*-sulfonated IAA (SIAA) in stinging nettle (*Urtica dioica*) which is a dioecious perennial herb. The putative identification of SIAA structure was based on the MS^2^ spectrum (Fig. 2) which contained two specific ions of [M-CO_2_-H]^−^ and [M-SO_3_-H]^−^, that correspond to the neutral loss of CO_2_ and SO_3_, respectively. Interestingly, the [M-SO_3_-H]^−^ ion was significantly less intense than that of *O*-sulfonated metabolites where the [M-SO_3_-H]^−^ fragment is usually the most intensive (Supikova, et al. 2022; Yi, et al. 2006). The lower intensity of [M-SO_3_-H]^−^ in the SIAA spectrum is likely due to an uneasy cleavage of the N-SO_3_H bond if compared to the *O*-sulfonated compounds, which is likely related to the differences in electronegativities of the S–O and S–N atoms (Yi, et al. 2006). The possible attachment of the sulfo group to the carbon in SIAA has also been considered. However, the only known natural *C*-sulfonated metabolite is the sulfolipid sulfoquinovosyl diacylglycerol, a component of chloroplast membranes, which is not even biosynthesized from PAPS like common *O*-sulfonated metabolites, but from sulfite anion (Sanda, et al. 2001). Therefore, the unknown structure was expected to contain an N-SO_3_H rather than C-SO_3_H moiety. To verify this assumption, a standard of *N*-sulfoindole-3-acetic acid (SIAA, 2-(1-sulfo-1H-indol-3-yl)acetic acid) was synthesized and its proposed structure was confirmed (Fig. S2 and S3). SIAA was subsequently analyzed in a broader range of species, e.g. *Arabidopsis*, wheat, barley and maize, using targeted methods (Tab. S2). Although SIAA was not detected in any of the other samples, suggesting that it may be a species-specific metabolite, it should be noted that the presence of SIAA in other plants cannot be completely excluded. In the targeted method, the LOD for SIAA was 24.4 fmol/injection which corresponds to 244 fmol/g DW. Therefore, if SIAA was present in the analyzed plants at concentrations below 244 fmol/g DW, it would not have been detected.

*N*-Sulfonated metabolites are rare in nature, and all of them, like SIAA, are *N*-sulfoindoles. The first two examples are *N*-sulfonyl-L-tryptophan (tryptorheedei A) and SILA (3-(*N*-sulfonylindolyl)-*D*-lactic acid/tryptorheedei B), which were identified in the seed kernels of the liana plant *Entada rheedei* (Nzowa, et al. 2013). The third and last known *N*-sulfometabolite from plants is glucobrassicin-1-sulfonate (1-sulfo-3-indolylmethylglucosinolate) which was identified in 1-week-old seedlings of woad (*Isatis tinctoria*)*,* a traditional source of indigo dye (Elliott and Stowe 1970). This glucosinolate contains two sulfogroups; one being attached to the isothiocyanate nitrogen forming the N–O-SO_3_H bond typical for glucosinolates, and another one attached directly to the nitrogen of the indole ring (N-SO_3_H) as in SIAA (Elliott and Stowe 1970).

Besides *N*-glucosylated IAA and its amino acid conjugates, SIAA represent another rare example of IAA modification via the nitrogen of the indole ring (Kai, et al. 2007). Other IAA metabolites and conjugates are mostly glycosyl esters, methyl esters and amide-linked conjugates, where the modifying moiety is localized on the acetic acid side chain, and their primary functions are storage and inactivation (Korasick, et al. 2013; Ludwig-Muller 2011). Another type of IAA modification is oxidation, which is typically located on the carbons of the indole ring, and results in the formation of oxIAA and 3-hydroxy-oxIAA (dioxIAA) (Hayashi, et al. 2021). In contrast to modifications located on the carboxyl group, this reaction is irreversible and represents IAA degradation (Brunoni, et al. 2023; Hayashi, et al. 2021; Porco, et al. 2016).

### Auxin-related sulfonated metabolites in *Urtica*

SILA, which was putatively identified in *Urtica* plants and seedlings, was previously reported also in *Entada rheedei* (Nzowa, et al. 2013). Similar to the sulfonation of IAA (Fig. 4), SILA may be biosynthesized from ILA, a weak auxin analog originating from Trp. However, there have been no reports of N-specific SOTs from plants to date (Günal, et al. 2019). ILA typically occurs in soil microorganisms such as bacteria (e.g. *Piriformospora indica* and duckweed-associated bacteria), fungi (e.g. *Neurospora crassa*), and less frequently in plants (e.g. tomato and grapevine) (Hilbert, et al. 2012; Sardar and Kempken 2018; Sprunck, et al. 1995). The metabolism of ILA includes its conversion to indole-3-pyruvic acid (IPyA) which is further metabolized to IAA in plants by YUCCA enzymes (Hilbert, et al. 2012; Sardar and Kempken 2018; Sprunck, et al. 1995; Zhao 2012). Thus, it might be possible that SILA is transformed to SIAA, like ILA to IAA, if relevant enzymes can accept sulfonated metabolite as a substrate in *Urtica*.

SPAA and SPLA, the derivatives of natural phytohormone PAA, were also identified in *Urtica* for the first time (Fig. 4). The non-sulfonated analogs, HPAA and HPLA may serve as substrates for unknown *Urtica* SOTs that may be responsible for the biosynthesis of SPAA and SPLA. Both HPAA and HPLA are naturally occurring metabolites; the first one is known mainly from bacteria and humans, and the second one is commonly present in plants (e.g. Alismatales, Maranthaceae, Liliales, Lamiaceae, Boraginaceae). Specifically, HPLA is biosynthesized from Tyr via 4-hydroxyphenylpyruvic acid, both of which are intermediates in the biosynthesis of rosmarinic acid (Abdullah, et al. 2008; Xu, et al. 2018), while HPAA is likely biosynthesized from 4-hydroxyphenylacetaldehyde by 4-hydroxyphenylacetaldehyde:NAD + oxidoreductase, although this enzyme was found only in *Pseudomonas putida* (Arcos, et al. 2010). In plants, namely in parsley, there was only confirmed the biosynthesis of 4-hydroxyphenylacetaldehyde from Tyr, but not its further conversion to HPAA (Torrens-Spence, et al. 2012).

### Sulfonated IAA has no auxin activity and effect on phenotype

SIAA had no auxin activity in the GUS assay (Fig. 5), which is consistent with the phenotype analysis (Fig. 8), where SIAA did not negatively affect the rosette size of *Arabidopsis* in contrary to IAA. The highest concentration of SIAA resulted in the slight increase in GUS activity, which may be due to the degradation of SIAA to IAA, enzymatic release of free IAA by sulfatases, or nonspecific binding of excess SIAA to the receptor. The same factors probably influenced the phenotype analysis (Fig. 8) where 1 μM SIAA caused a slight inhibition of rosette leaf growth; however, this effect was still less pronounced than that observed with 0.1 μM IAA. The negative effect of IAA on rosette size and leaf number in *Arabidopsis* was previously observed, e.g. in superroot mutants (*sur1—7*) with elevated endogenous IAA levels and in *Arabidopsis* treated with exogenous IAA (Boerjan, et al. 1995). Furthermore, (2-sulfoindole)-3-acetic acid, a synthetic derivative of IAA with the sulfo group attached to the carbon C2, showed no auxin activity in an assay with mung bean hypocotyls (Horng and Yang 1975; Lau, et al. 1978). As suggested by these results, sulfonation of IAA inactivates its biological activity; thus, it may regulate auxin signaling in *Urtica*. However, the reversibility of sulfonation in general has not been confirmed (Günal, et al. 2019). Sulfonation may also facilitate IAA transport in the apoplast as they increase the polarity and solubility of molecules (Günal, et al. 2019; Kurth, et al. 2015; Yi, et al. 2006). Another function of SIAA may be the storage of excess sulfur in *Urtica* as supposed for sulfonated flavonoids in *Flaveria* spp. (Kleinenkuhnen, et al. 2019).

Inactivation of the physiological activity of phytohormones by sulfonation has already been demonstrated for JA in plants (Fernández-Milmanda, et al. 2020; Gidda, et al. 2003; Miersch, et al. 2008). Specifically, 12-hydroxy-JA was sulfonated by *At*SOT15 during shade avoidance syndrome, forming inactive HSO_4_-JA and thereby inhibiting JA functions (Fernández-Milmanda, et al. 2020). Several other plant SOTs have been identified and cloned; some have shown specificity for other phytohormones. For example, *At*SOT12 was specific for SA and 24-epibrassinosteroids, and *At*SOT10 was specific for brassinosteroids, including (22R, 23R)-28-homobrassinosteroids and 24-epibrassinosteroids (Baek, et al. 2010; Marsolais, et al. 2007). Sulfonation of brassinosteroids resulted in the inhibition of their biological activity as shown in the assay of bean second internode (Rouleau, et al. 1999). However, sulfonated products of these two SOTs have only been prepared in vitro, and their presence in plants has not been confirmed (Baek, et al. 2010; Lacomme and Roby 1996; Marsolais, et al. 2007). The only phytohormones whose active form is sulfonated are phytosulfokines that naturally occur in all plants (Matsubayashi and Sakagami 1996).

### SIAA is biosynthesized from inorganic sulfate de novo in plants

The biosynthesis of SIAA de novo in *Urtica* seedlings was successfully demonstrated by the incorporation of ^34^S from exogenous ammonium sulfate labeled with stable ^34^S isotope (Fig. 6). The same was confirmed for SPLA and SILA, but not for SPAA, which may be due to its low tissue concentration or slower biosynthesis. In addition, since SPAA was not detected in *Urtica* seeds, it is likely to be biosynthesized at a later developmental stages but not in seedlings. A similar incorporation study was previously performed in *Arabidopsis* seedlings, to investigate the biosynthesis of all sulfur-containing metabolites (Gläser, et al. 2014). A combination of the feeding study with labeled ^34^S-sulfate and high-resolution MS (ion cyclotron resonance with Fourier transformation MS) resulted in the detection of approximately 140 unknown sulfur-containing metabolites (Gläser, et al. 2014). The identified metabolites included both, sulfonated secondary metabolites (e.g. quercetin 3,3'-bissulfate and neoglucobrassicin) and thiols predominantly involved in primary metabolism (e.g. glutathione, S-ribosyl homocysteine and CoA) (Gläser, et al. 2014).

The initial stage of sulfur assimilation always involves the conversion of inorganic exogenous ^34^S-sulfate to the organic form of ^34^S-adenosine-5'-phosphosulfate, and its further phosphorylation to ^34^S-PAPS (Hirschmann, et al. 2014). PAPS was also detected in the feeding study by Gläser et al. (2014) as a positively charged ion. In *Urtica* plants, ^34^S-sulfate was probably directly transferred from ^34^S-PAPS to IAA and/or its precursor such as ILA. Their sulfonation may be mediated by unknown SOT capable of transferring sulfate to the nitrogen of amines or indoles, although such enzymes have only been found in animals to date (Hirschmann, et al. 2014).

### SIAA is a main IAA metabolite in *Urtica* and is positively affected by blue light

SIAA was the most abundant IAA metabolite detected in *Urtica,* with concentrations being 27–162 times higher than those of IAA; however, the SIAA/IAA ratio showed significant differences when different light conditions were applied to *Urtica* plants (Fig. 7a; Tab. S3). Etiolated plants showed accumulated auxin levels due to the support of elongation growth, which is a known mechanism of photomorphogenesis (Halliday and Fankhauser 2003). In B and R light-grown plants, IAA concentrations were approximately 3 times reduced if compared to the dark-developed plants (Fig. 7a; Tab. S3), while SIAA was increased or remained the same in B and R light, respectively. Thus, the SIAA/IAA ratio was affected the most in B light, which is known to be involved in e.g. phototropins-mediated phototropism, chloroplast movements, leaf expansion and leaf movements, as well as cryptochromes-mediated repression of photomorphogenesis (Keuskamp, et al. 2010).

An important defense hormone, JA, also occurs in plants in both, sulfonated and non-sulfonated forms, whose ratio is modulated by light conditions. Particularly, during the shade avoidance syndrome in *Arabidopsis*, a low R:FR ratio, recognized via the photoreceptor phytochrome B, induces the expression of *At*SOT15 (ST2a), and the above-mentioned sulfonation of hydroxy-JA to HSO_4_-JA. This results in the decrease in active JA levels, and subsequent reduction in JA signaling. Thus, the defense response is attenuated while the growth is stimulated, which is a strategic behavior of plants lacking light (Fernández-Milmanda, et al. 2020; Gidda, et al. 2003).

Although the specific impact of different light conditions on IAA sulfonation is different from that reported for JA sulfonation, obviously due to their contradicting effects on growth/defense, the general mechanism involving activation of photoreceptors followed by light-induced expression of the putative sulfotransferase specific to IAA can be similar to that proposed for JA and HSO_4_-JA in *Arabidopsis* (Fernández-Milmanda, et al. 2020; Gidda, et al. 2003).

## Conclusion

Although *N*-sulfonated IAA has only been discovered in a single species, it represents a previously unknown auxin modification which may also be active in other plants in addition to *Urtica*. SIAA is not only a novel but also unique metabolite due to its *N*-sulfoindole substructure, which has only been reported in two previous studies (Elliott and Stowe 1970; Nzowa, et al. 2013). According to the results presented in this study, sulfonation of IAA at *N*-position eliminates its auxin activity, as shown by the DR5::GUS assay and phenotype analysis. Like other IAA conjugates, SIAA could serve as a storage form of IAA, although the reversibility of IAA sulfonation is uncertain. Sulfonation in plants is an exciting metabolic modification, which result in hundreds of sulfometabolites with poorly understood functions. The targets of this reaction are, besides secondary metabolites, growth regulators and phytohormones, which might directly or indirectly modulate growth and development *in planta*.

## Supplementary Information

Below is the link to the electronic supplementary material.

Supplementary file1 (DOCX 284 KB)

Supplementary file2 (DOCX 72 KB)

Supplementary file3 (DOCX 75 KB)

Supplementary file4 (DOCX 112 KB)

Supplementary file5 (XLSX 51 KB)

Supplementary file6 (XLSX 17 KB)

Supplementary file7 (XLSX 39 KB)

## Data Availability

The data from this study can be requested from the corresponding author.

## Abbreviations

ANT: Anthranilic acid
AtSOTs: Arabidopsis thaliana sulfotransferases
ARF: Auxin response factor
B: Blue
BPI: Base peak ion
DAO1: Dioxygenase for auxin oxidation 1
dioxIAA: 3-Hydroxyoxindole-3-acetic acid
DW: Dry weight
EDTA: Ethylenediaminetetraacetic acid
ESI: Electrospray ionization
EtOAc: Ethyl acetate
EtOH: Ethanol
FR: Far red
FW: Fresh weight
Glc: Glucose
HPAA: 4-Hydroxyphenylacetic acid
HPLA: 4-Hydroxyphenyllactic acid
HSO4-JA: 12-(Sulfooxy)jasmonic acid
IAA: Indole-3-acetic acid
IAM: Indole-3-acetamide
ILA: Indole-3-lactic acid
IPyA: Indole-3-pyruvic acid
JA: Jasmonic acid
LC: Liquid chromatography
MeOH: Methanol
MRM: Multiple reactions monitoring
MS: Mass spectrometry
MS/MS: Tandem mass spectrometry
m/z: Mass-to-charge ratio
ND: Not detected
NMR: Nuclear magnetic resonance
oxIAA: Oxindole-3-acetic acid
PAA: Phenylacetic acid
PAPS: 3'-Phosphoadenosine-5'-phosphosulfate
QqQ: Triple quadrupole mass analyzer
QqTOF: Quadrupole time-of-flight mass analyzer
R: Red
RT: Retention time
S/N: Signal-to-noise ratio
SD: Standard deviation
SIAA: *N*-Sulfoindole-3-acetic acid
SILA: *N*-Sulfoindole-3-lactic acid
SOTs: Sulfotransferases
SPAA: 4-(Sulfooxy)phenylacetic acid
SPLA: 4-(Sulfooxy)phenyllactic acid
TPSTs: Tyrosylprotein sulfotransferases
TRA: Tryptamine
TRP: Tryptophan
UHPLC: Ultra-high-performance liquid chromatography
W: White

## Footnotes

1 The terms "sulfonation" and "sulfonated" are used throughout the text to generally describe the enzymatic transfer of -SO_3_H to the substrate molecule, although "sulfation" and "sulfated" are more commonly used in the literature, especially to describe metabolites containing the -OSO_3_H group (sulfate).
