# Supplementary material for: Sulfonation of IAA in Urtica eliminates its DR5 auxin activity
Source: Plant Cell Rep. 2024 Dec 20;44(1):8. doi: 10.1007/s00299-024-03399-1 (PMC11662057; doi:10.1007/s00299-024-03399-1)
Supplement: Supplementary file 1 — Supplementary file1 (DOCX 284 KB) [file 299_2024_3399_MOESM1_ESM.docx]

**A)**

**B)**

**Figure S1** ^1^H NMR (A) and ^13^C NMR (B) spectra of SIAA.
