# Supplementary material for: Sulfonation of IAA in Urtica eliminates its DR5 auxin activity
Source: Plant Cell Rep. 2024 Dec 20;44(1):8. doi: 10.1007/s00299-024-03399-1 (PMC11662057; doi:10.1007/s00299-024-03399-1)
Supplement: Supplementary file 2 — Supplementary file2 (DOCX 72 KB) [file 299_2024_3399_MOESM2_ESM.docx]

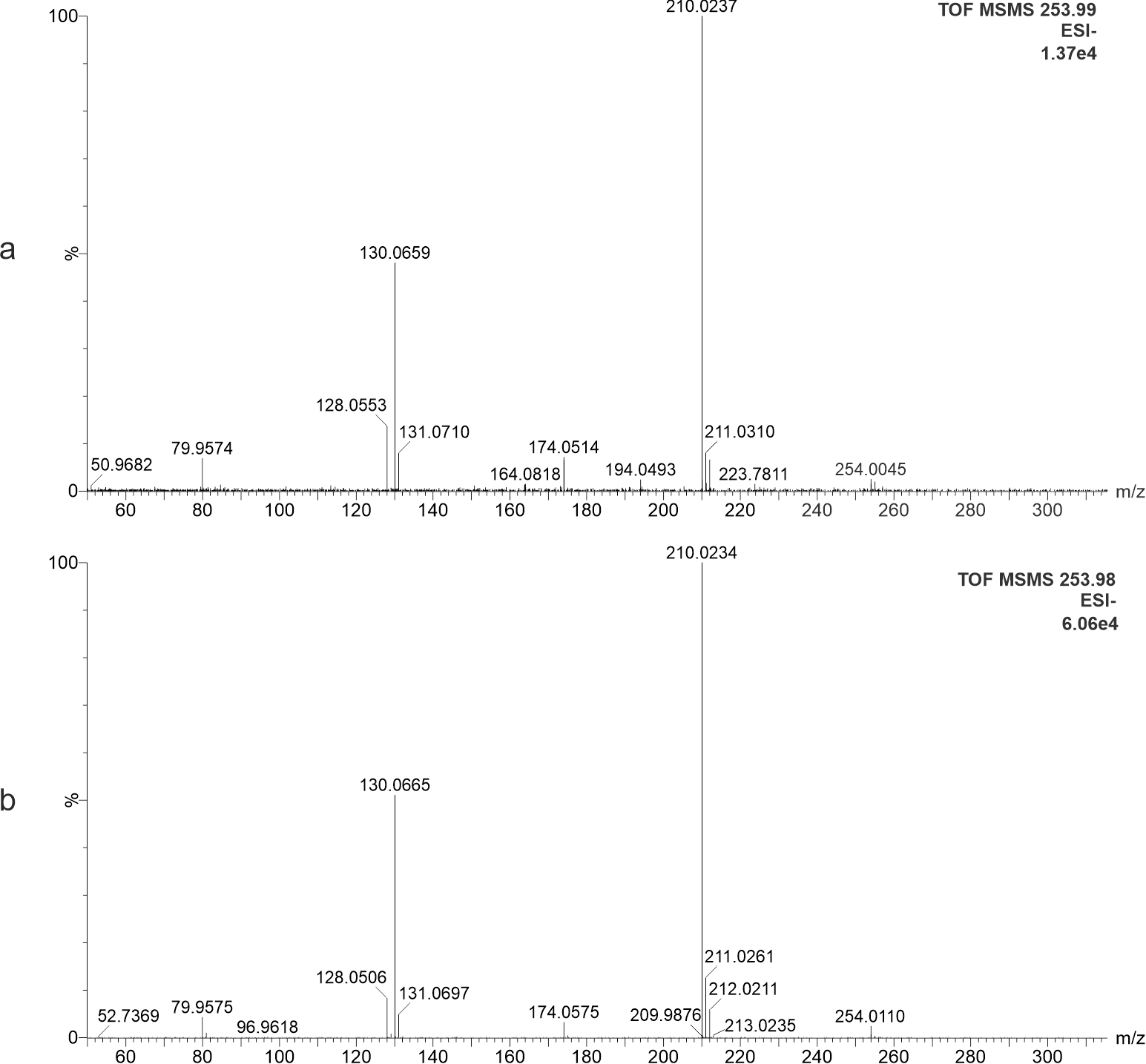


**Figure S2 a** MS/MS spectrum of *N*-sulfoidole-3-acetic acid (SIAA) from *Urtica dioica* extract detected by UHPLC-QqTOF-MS analysis. The collision energy was -20 eV. **b** MS/MS spectrum of synthetic standard of SIAA analyzed by UHPLC-QqTOF-MS analysis. The collision energy was -20 eV.
