# Supplementary material for: Sulfonation of IAA in Urtica eliminates its DR5 auxin activity
Source: Plant Cell Rep. 2024 Dec 20;44(1):8. doi: 10.1007/s00299-024-03399-1 (PMC11662057; doi:10.1007/s00299-024-03399-1)
Supplement: Supplementary file 3 — Supplementary file3 (DOCX 75 KB) [file 299_2024_3399_MOESM3_ESM.docx]

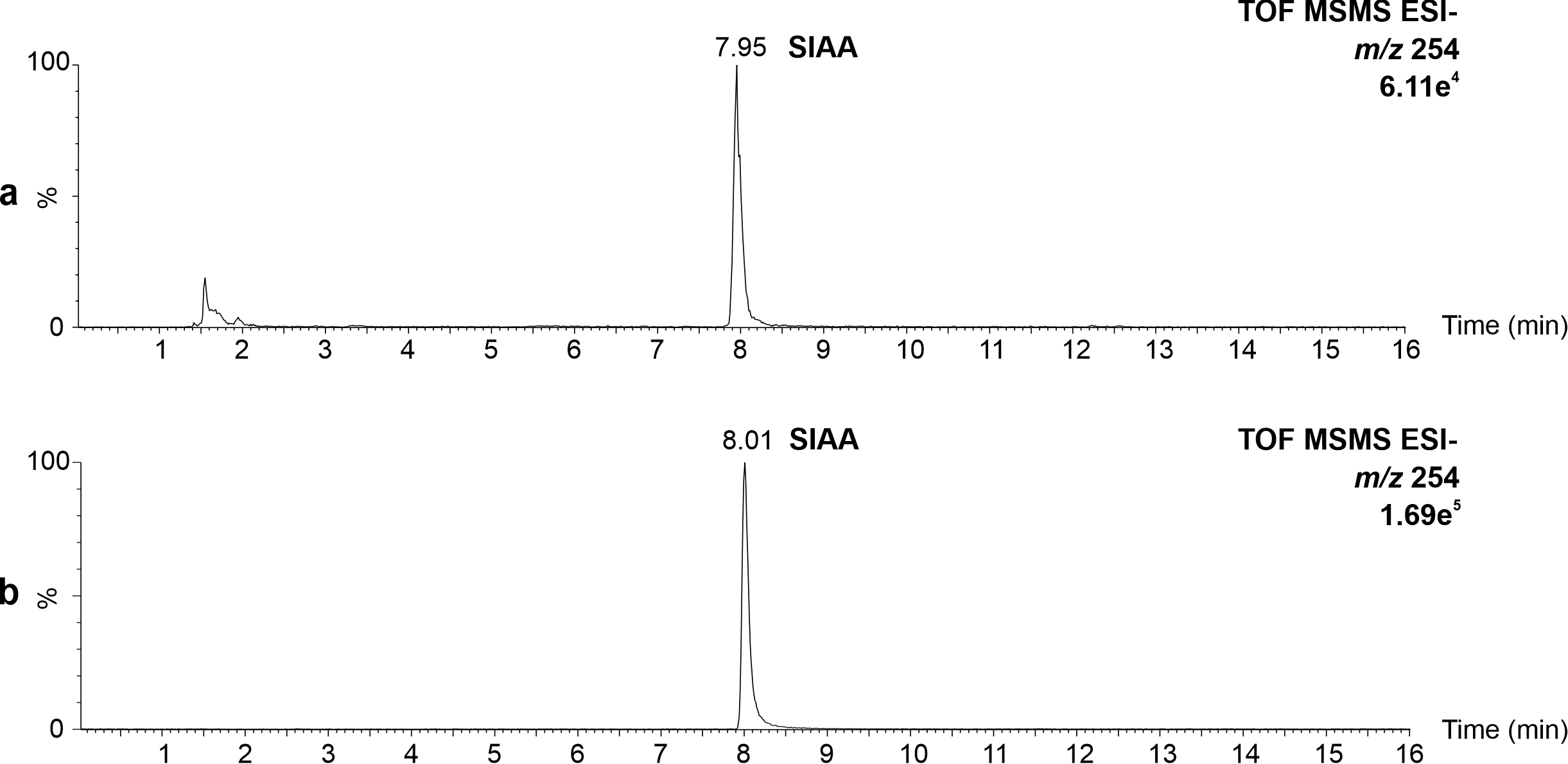


**Figure S3 a** Chromatogram of SIAA detected in an extract of *Urtica dioica* by UHPLC-QqTOF-MS analysis. **b** Chromatogram of synthetic standard of SIAA (1·10^-5^ M) analyzed by UHPLC-QqTOF-MS analysis.
