# Supplementary material for: Sulfonation of IAA in Urtica eliminates its DR5 auxin activity
Source: Plant Cell Rep. 2024 Dec 20;44(1):8. doi: 10.1007/s00299-024-03399-1 (PMC11662057; doi:10.1007/s00299-024-03399-1)
Supplement: Supplementary file 4 — Supplementary file4 (DOCX 112 KB) [file 299_2024_3399_MOESM4_ESM.docx]

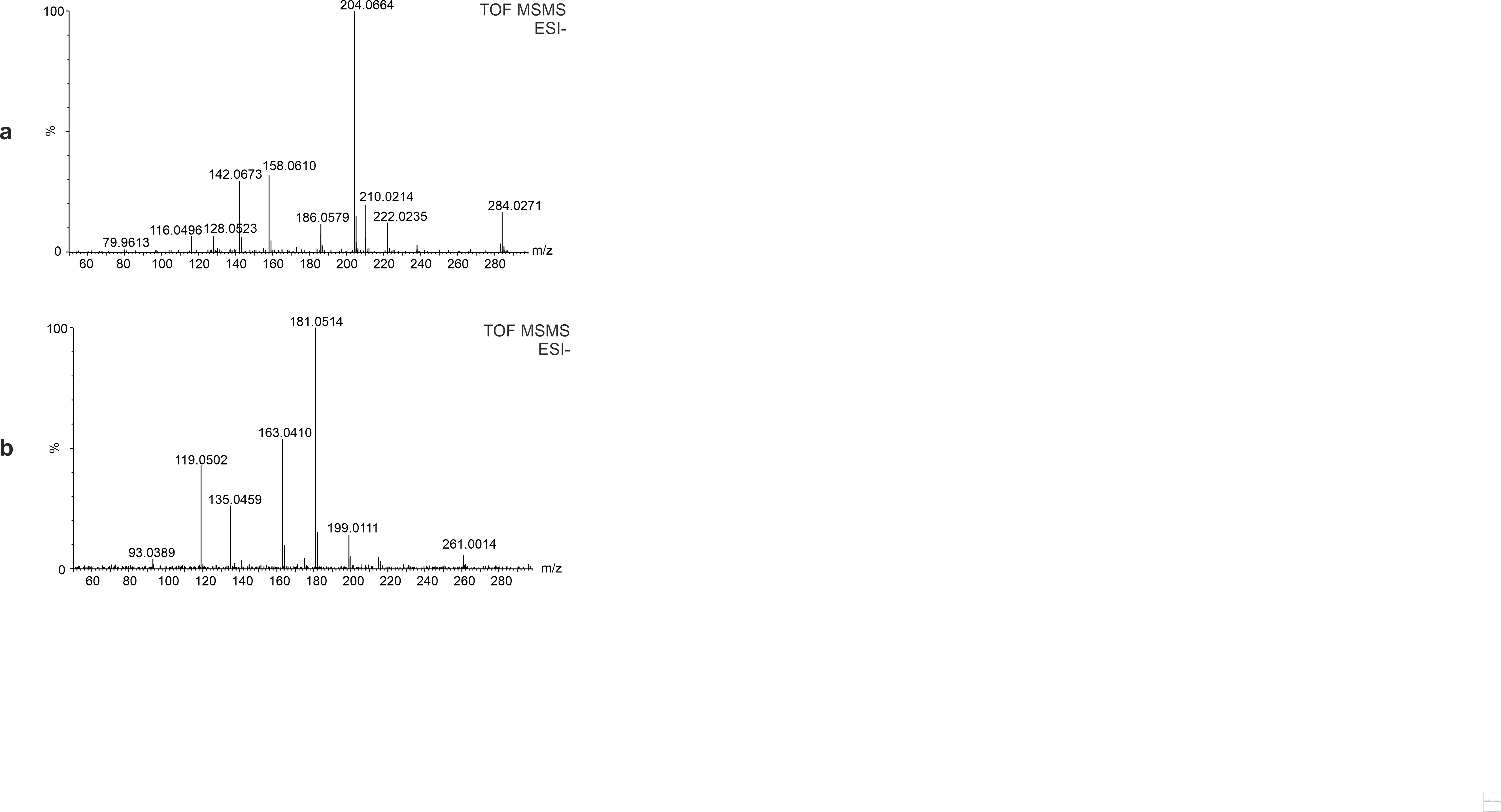


**Figure S4 a** MS/MS spectrum of *N*-sulfoindole-3-lactic acid (SILA) with *m/z* 284.0271 acquired by UHPLC-QqTOF-MS analysis. The collision energy was -20 eV. **b** MS/MS spectrum of 4‑(sulfooxy)phenyllactic acid with *m/z* 261.0014 acquired by UHPLC-QqTOF-MS. The collision energy was -20 eV.
